# A Drug Content, Stability Analysis, and Qualitative Assessment of Pharmacists’ Opinions of Two Exemplar Extemporaneous Formulations

**DOI:** 10.3390/molecules25133078

**Published:** 2020-07-06

**Authors:** Melissa Kirkby, Kurtis Moffatt, Aoife M. Rogers, Paul J. McCague, James C. McElnay, Caoimhe Quinn, Lezley Ann McCullough, Johanne Barry, Ryan F. Donnelly

**Affiliations:** School of Pharmacy, Medical Biology Centre, Queens University Belfast, 97 Lisburn Road, Belfast BT9 7BL, UK; m.kirkby@qub.ac.uk (M.K.); k.moffatt@qub.ac.uk (K.M.); a.rogers@qub.ac.uk (A.M.R.); p.mccague@qub.ac.uk (P.J.M.); j.mcelnay@qub.ac.uk (J.C.M.); cquinn80@qub.ac.uk (C.Q.); lmccullough01@qub.ac.uk (L.A.M.); johanne.barry@qub.ac.uk (J.B.)

**Keywords:** community pharmacist, extemporaneously compounding, omeprazole, amlodipine, unlicensed

## Abstract

Despite a decline in the number of active pharmaceutical ingredients prepared extemporaneously using proprietary products, there remains a need for such products in the community (for example, liquid medicines for paediatrics which may be otherwise commercially unavailable). A lack of experience and quality assurance systems may have diminished pharmacist’s confidence in the extemporaneous preparation process; therefore, pharmacists were asked to prepare two proprietary products, omeprazole and amlodipine. The resulting products were characterised in terms of variability in drug quantity, stability, particle size and antimicrobial properties. Furthermore, a self-administered questionnaire was used to assess 10 pharmacists’ opinions on the perceived complexity of the extemporaneous compounding process and their overall confidence in the final extemporaneously compounded products. Drug content studies revealed that 88.5% and 98.0% of the desired drug content was obtained for omeprazole and amlodipine, respectively. Antimicrobial properties were maintained for both drugs, however variability in particle size, particularly for amlodipine, was evident between formulations. While pharmacists who partook in the study had some or high confidence in the final products, they reported difficulty formulating the suspensions. Findings from this study provide insight into pharmacists’ views on two extemporaneously prepared products and highlight the variability obtained in preparations prepared by different pharmacists.

## 1. Introduction

Extemporaneous compounding describes the process in which medicines are prepared in a bespoke manner, by a pharmacist or by a suitable individual under pharmacist supervision, for supply in accordance with a prescription on a named patient basis [1]. Extemporaneously compounded medicines are typically required to meet a specific patient need in circumstances such as paediatrics, for those who cannot swallow tablets, where the dose required is unavailable in solid dosage forms, or for patients with naso-gastric feeding tubes in place [2].

The ability to dispense medicines extemporaneously has long been considered as a skill uniquely attributable to the pharmacy profession [3]. Since the tragic peppermint water case [4], concerns have been raised regarding extemporaneously dispensed medicines and subsequent patient safety issues [5]. Conroy has previously reported the difficulties faced when prescribing, dispensing, or administering medicines to children [6]. A lack of information and suitable formulations leads to the need to use unlicensed and off-label products on a regular basis.

It has been proposed that risks associated with extemporaneously compounded medicines have increased due to a decreasing expertise in the formulation of pharmaceutics within the profession as a result of reduced prescribing of these types of medicines and as pharmacists move towards a clinically-focused approach [7]. In support of this proposal, a letter in The Pharmaceutical Journal states that pharmacists may not encounter requests for extemporaneously compounded products often enough to maintain and develop their confidence in this area of practice [8].

Pharmacists are often hesitant to dispense extemporaneously compounded products because of lack of confidence in their final product. This is due to several reasons, in addition to the argument proposed by Neill [8], including that extemporaneous preparations carry additional risks of non-standardised formulations, potential formulation failure, possible microbiological contamination and lack of stability information. As a result, there has recently been an increase in the use of “specials” by pharmacists as an alternative [9], though many pharmacists would like extemporaneous dispending to be retained by the profession [10].

Despite a decreased frequency in the number of extemporaneously compounded products, they still represent a significant number of products, indicating a continued requirement for this service in the community. Thus, there is a requirement to assess the ability of pharmacists in the extemporaneous compounding process and to evaluate their own confidence in their skills to do so. This study aimed to explore pharmacists’ confidence in extemporaneous compounding and assess the final extemporaneously compounded products for quantification of drug content, stability analysis and antimicrobial preservation. Amlodipine (5 mg tablets) and omeprazole (20 mg capsules) were selected as two exemplar extemporaneously compounded products, as these are commonly dispensed for paediatric patients by community pharmacists [11].

The results from this study demonstrate the variability in extemporaneously prepared compounds from different pharmacists. Whilst there was no significant difference the stability, and both formulations passed the British Pharmacopoeia (BP) efficacy of antimicrobial preservative tests, there were clear variabilities in particle size. This may give rise to inconsistencies in dosing due to sedimentation of the drug and variance in drug absorption. Furthermore, pharmacists who partook in the study reported some or high confidence in the final extemporaneously compounded products, though they reported difficulty formulating the omeprazole suspension, attributed to the difficulty in crushing the enteric-coated pellets.

Dosing inconsistencies from extemporaneously prepared products increases the risk of either drug overdose or sub-therapeutic dosing. The lack of guidance on acceptable parameters specifically for extemporaneously prepared products suggests that clearer criteria from governing bodies, such as the Pharmaceutical Society of Northern Ireland (PSNI), may be required, alongside continual revalidation to allow pharmacists to realistically gauge whether their extemporaneously produced product is suitable for release to the patient. Furthermore, more licensed products may be required to ensure optimal pharmacological therapy, particularly for paediatric patients where the therapeutic window is narrow.

## 2. Results

### 2.1. Pharmaceutical Analysis of Omeprazole and Amlodipine

The calibration curve properties for the HPLC analytical method, including the limit of detection (LoD) and limit of quantification (LoQ) for omeprazole and amlodipine, is displayed in Table 1. The retention time for omeprazole and amlodipine was found to be approximately 1.8 and 9.6 min, respectively.

### 2.2. Preparation of Formulations of Omeprazole and Amlodipine by Pharmacists: Drug Content, Stability Studies and Analysis of Particle Size Distribution

As requested, all ten pharmacists who participated in the study prepared the two formulations of omeprazole and amlodipine in a timely manner over the space of forty-five minutes. However, it was observed that one pharmacist incorrectly used water for the suspension of the crushed amlodipine tablets rather than the suspending agent provided. No other errors in the preparation of the formulations were observed or self-reported by the pharmacists.

Following the formulation of omeprazole and amlodipine, subsequent drug stability and stability profile studies were conducted to assess the variability in the prepared formulations. Initial drug content was calculated based on the peak area post-injection of the formulation following the extraction procedure. Results for the omeprazole and amlodipine suspensions as prepared by the ten pharmacists are shown in Table 2. The target concentration for the omeprazole suspension was 2 mg/mL. The mean concentration obtained by pharmacists was 1.77 mg/mL (± 0.18 mg/mL). The drug content ranged from 62.84% to 93.74% of that expected. The target concentration for the amlodipine suspension was 1.00 mg/mL. The mean concentration achieved was 0.98 mg/mL (± 0.16 mg/mL) and the percentage drug content ranged from 72.63% to 120.90%.

**Figure 1:**
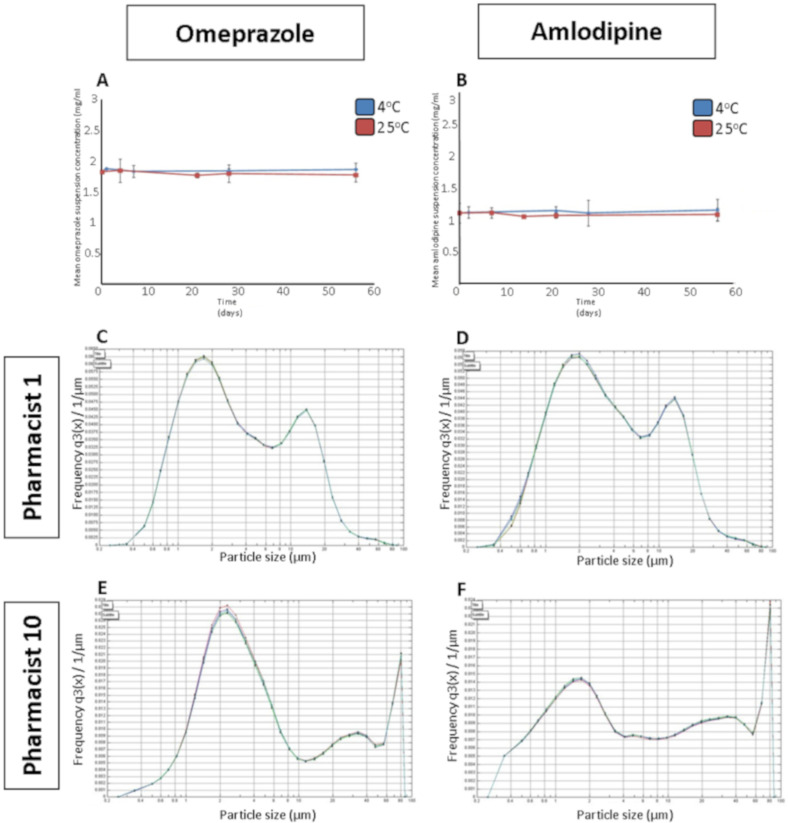
The stability profile of extemporaneously prepared (**A**) omeprazole and (**B**) amlodipine suspensions over a 56-day period stored at 4 °C and 25 °C. Statistical significance was determined using a Wilcoxon Signed Rank test, *n* = 5. Differences in the particle size distribution of suspensions prepared by pharmacist 1: (**C**) omeprazole, (**D**) amlodipine, and pharmacist 10: (**E**) omeprazole, (**F**) amlodipine.

Figure 1 details the characterisation of both omeprazole and amlodipine in terms of stability profile and particle size. Figure 1A,B illustrate the stability profile of omeprazole and amlodipine, respectively, when the two formulations were stored at room temperature (25 °C) and in a refrigerator (4 °C) over a 56-day period. No statistical significance was obtained between the concentrations of an extemporaneously prepared omeprazole suspension between day 0 and day 56, when stored at 4 °C (*p* = 0.08) or at 25 °C (*p* = 0.08). Similarly, a Wilcoxon Signed Rank test shows that there was no significant difference between the concentration of an extemporaneously prepared amlodipine suspension between day 0 and day 56 when stored at 4 °C (*p* = 0.08) or at 25 °C (*p* = 0.22).

Figure 1C–F illustrates the variation in particle size of two omeprazole (1C and 1E) and amlodipine (1D–F) formulations when each are prepared by two different pharmacists, Pharmacist 1 and Pharmacist 10. Five replicates were analysed for each formulation and measured over the range of 0.2–85 μm. As evident, there is variation in the particle size distribution of the omeprazole suspension prepared by Pharmacist 1 and 10. Moreover, a similar result was obtained for the amlodipine suspensions (Figure 1D,F), whereby variation in the particle size of the suspensions prepared by Pharmacist 1 and Pharmacist 10 is clearly evident. A greater proportion of the particles in the amlodipine suspension were between 1.0–6.0 μm for Pharmacist 1 compared to Pharmacist 10. Less variation between the two pharmacists’ formulations is evident for the omeprazole formulations compared to the amlodipine formulations.

### 2.3. Assessing Efficacy of Antimicrobial Preservation

Table 3 shows a viable count taken for each formulation at day 0, representing the initial challenge of bacteria to each formulation sample and the subsequent log reduction of bioburden over the 28 day study period. As shown, there was a greater than three-log reduction in bacterial load after 14 days and no increase in bioburden was evident after 28 days. Thus, both formulations passed the BP efficacy of antimicrobial preservative for the bacterial species tested.

### 2.4. Assessing Pharmacists’ Opinions

All pharmacists (*n* = 10) completed and returned the survey. The ten participants consisted of five female and five male pharmacists; all had a background in community and/or hospital pharmacy. The pharmacists were first asked whether or not they had prepared the particular formulation in the past. Four pharmacists had previous experience preparing omeprazole suspensions in the past, whilst one pharmacist had previously prepared the amlodipine suspension. Pharmacists were asked to rate their overall confidence in the final product and responses were scaled 1–5, where 1 = no confidence and 5 = very confident.

**Figure 2:**
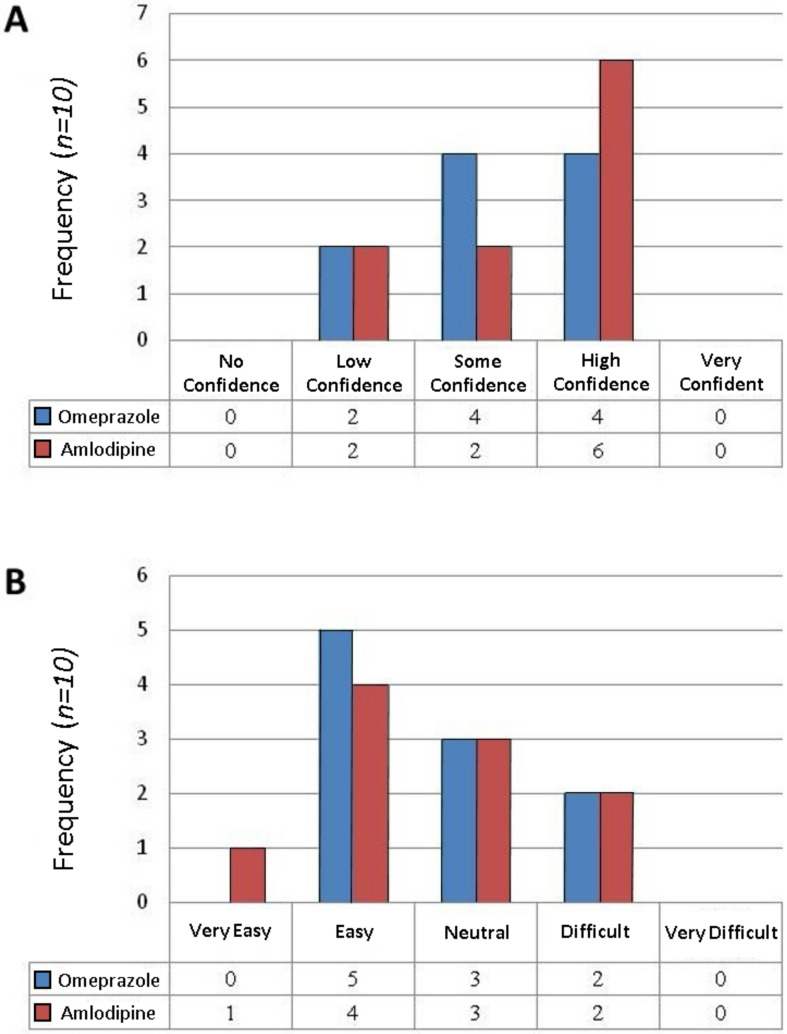
A bar chart showing pharmacists’ level of confidence in the final preparation of omeprazole and amlodipine (**A**) and the level of complexity preparing the product (**B**).

Figure 2A shows the pharmacists’ level of confidence in the final preparations of omeprazole and amlodipine. The median score was 3.00 (some confidence) for the omeprazole suspension and 4.00 (high confidence) for the amlodipine suspension. Thus, there were no significant difference in the pharmacists’ level of confidence in the final preparation (*p* > 0.05). Pharmacists were asked to rate the complexity of preparing the two formulations. Responses were scaled 1–5 where 1 = very easy to 5 = very difficult. Results are presented in Figure 2B. The median score was 2.50 (neutral) for both suspensions, resulting in no statistical significance (*p* > 0.05) in pharmacists’ perceived level of complexity in comparing the two products (Table 4).

Many pharmacists commented on the challenges experienced while formulating the omeprazole products, particularly with respect to crushing the enteric coated granules.

‘…crushing granules was difficult and they kept escaping from the mortar’.

(Pharmacist 10)

‘Extremely time consuming due to the relative difficulty in grinding/crushing the small and hard granules within the capsule’.

(Pharmacist 5)

‘It was likely some of the active ingredient was lost during the crushing process due to the size of the granules within each capsule’.

(Pharmacist 2)

Pharmacists appeared to find the amlodipine suspension less challenging to formulate:

‘Tablets were easier to crush than the omeprazole granules’.

(Pharmacist 5)

‘Tablets were more easily crushed in this case’.

(Pharmacist 4)

Pharmacists found preparation of both formulations a time-consuming task:

‘Crushing the tablets uniformly was time-consuming’.

(Pharmacist 10)

To summarise, drug content studies revealed that 88.5% and 98.0% of the desired drug content was obtained for the omeprazole and amlodipine suspensions, respectively, with no significant degradation of either drug over a 56-day period when stored at room temperature or in a refrigerator. Whilst both formulations passed the BP test for antimicrobial preservative, there was a wide range of particle sizes reported within the suspensions prepared by different pharmacists. Pharmacists had ‘some confidence’ in the quality of the omeprazole formulation and ‘high confidence’ in the amlodipine suspension. The main difficulty associated with formulating the omeprazole suspension was crushing the enteric-coated pellets in a uniform manner without their escape for the mortar.

## 3. Discussion

### 3.1. Preparation and Characterisation of Omeprazole and Amlodipine Formulations

Despite a decline in the number of extemporaneously compounded products dispensed in recent years, largely due to a sub-contract to specials manufacturers, there remains a substantial number dispensed in the UK every year, with only the pharmacist capable of delivering such specialised medicinal products. Thus, pharmacists’ skills and knowledge to prepare such formulations must remain at a high standard to ensure efficacy in the final extemporaneously compounded products, but assurance of patient safety is also paramount [10,12]. Accordingly, using two exemplar active pharmaceutical ingredients, this study assessed pharmacists’ ability and self-confidence in the extemporaneous compounding process.

Analysis of the final extemporaneously compounded products’ drug content revealed variable results between participants. The target concentration for the omeprazole and amlodipine suspension was 2 mg/mL and 1 mg/mL, respectively. Approximately 88.5% and 98.0% of the desired drug content was obtained. The PSNI and the General Pharmaceutical Council (GPhC) do not provide any guidance as to what concentration range of active ingredient is acceptable for extemporaneously prepared products. Professional guidance published by the PSNI in 2016 states: ‘patients are entitled to expect that products extemporaneously prepared in a pharmacy are prepared accurately and suitable for use’ [13]. However, the PSNI do not quantify or define ‘accurately’. The GPhC provided a “five step” risk assessment to manage the risks of preparing the extemporaneous products themselves [14], though this guidance centred on safeguarding patients rather than the actual preparation of these products. The BP monographs of marketed formulations state that the drugs should be present in the formulations within a 95–105% range of the stated content. Of the formulations prepared by the ten pharmacists herein, only two amlodipine suspensions were formulated within this concentration range, while none of the omeprazole suspensions reached this standard. Ideally, when extemporaneously compounding, pharmacists should prepare drugs in accordance with a BP monograph where available [15]. However, it is recognised that the two products do not have BP monographs and therefore no guidelines exist on the concentration ranges of the active ingredients of the products.

Rosemont Pharmaceuticals, a Medicines and Healthcare products Regulatory Agency (MHRA) licensed specials manufacturer, is an example of one of the commercial specials manufacturers in the UK. It complies with quality assurance (QA) and quality control (QC) guidelines and products are formulated within a 95–105% concentration range [16]. Community and hospital pharmacies do not however have the same equipment or capabilities of ensuring QA or QC as an industrial facility. Another difficulty in achieving a concentration of 95–105% is the fact that these formulations were prepared using licensed products, which themselves have an accuracy of 95–105%. So, if the omeprazole capsules and/or amlodipine tablets had an initial strength of 95% of the target strength, this has already increased the potential for significantly lowering the drug content before the pharmacist even begins to formulate the product.

One pharmacist who participated in the study ‘suspended’ the amlodipine tablets in water rather than the suspending agent, highlighting the errors which can occur when extemporaneously preparing medicines. This resulted in a product that was unusable, as the amlodipine would not suspend in the water and lacked a preservative. There could be serious consequences had this extemporaneously compounded product been dispensed for patient use. Assessment of chemical stability indicated that potency was maintained for the two exemplar extemporaneously compounded suspensions between day 0 and day 56 when stored at 4 °C or at 25 °C, although the shelf-life for both these products is stipulated at 28 days.

Particle size analysis results demonstrated that a wide range of particle sizes exist within the suspensions and indeed can vary between suspensions of the same formulation prepared by different participants. Such variations in particle size could give rise to inconsistencies in dosing due to sedimentation of the drug and indeed variance in absorption of the drug *in vivo*. A lack of appropriate shaking before use can greatly affect particle size within a suspension; a study by Saidum and Pratheepawanit found that despite the fact that around two-thirds of participants were aware of the correct way to shake a bottle before pouring a medicine, only 12% of parents were able to shake the medicine properly [17]. Furthermore, when left undisturbed for a long period of time, the particles in the suspension will aggregate, sediment, and eventually cake. Thus, thorough shaking is essential prior to administration of a suspension to improve dose reliability [18].

Both formulations passed the BP efficacy of antimicrobial preservative for the bacterial species tested, as there was a greater than three-log reduction in bacterial load after 14 days and no increase in bioburden after 28 days. In the case of omeprazole, there is no recognised preservative, but the suspending agent used is sodium bicarbonate (8.4%), which has been previously reported to have antimicrobial properties [19] due to its alkaline nature. The suspending agent used to formulate the amlodipine suspension (Orablend^®^) contains preservatives, including methylparaben, propylparaben and potassium sorbate. Methylparaben exhibits antimicrobial activity at pH 4–8. Parabens are more active against Gram-positive bacteria than against Gram-negative bacteria. Methylparaben is the least active of the parabens; antimicrobial activity increases with increasing chain length of the alkyl moiety. Activity may be improved by using combinations of parabens as synergistic effects occur [20]. Potassium sorbate has both antifungal and antibacterial properties. The antimicrobial activity is dependent on the degree of dissociation; there is practically no antibacterial activity above pH 6. The efficacy of potassium sorbate is also increased when used in combination with other antimicrobial preservatives, such as parabens, since synergistic effects occur [20].

### 3.2. Pharmacists’ Opinions and Confidence Surrounding the Perceived Complexity of the Extemporaneous Compounding Process

Pharmacists must also exhibit confidence in their skill and in the final extemporaneously compounded product. Analysis of the survey results showed the median score was 3.00 (some confidence) for the omeprazole suspension and 4.00 (high confidence) for the amlodipine suspension. However, there was no significant difference between pharmacists’ confidence in the two products. This is comparable to a survey of 420 pharmacists carried out in 1999, which indicated that 68% of respondents had high or very high confidence in their extemporaneous compounding ability [10]. Free-text responses showed that many of the pharmacists reported difficulty formulating the omeprazole suspension (in particular, difficulty crushing the enteric-coated pellets and retaining the pellets in the mortar). This may explain why a lower concentration of drug was observed in the omeprazole suspensions. Given such results, it is understandable that pharmacists lack confidence in the preparation of such formulations.

## 4. Materials and Methods

### 4.1. Chemicals

Amlodipine besilate and omeprazole were obtained from Tokyo Chemical Industry Ltd., Oxford, UK. Amlodipine besilate (5 mg tablets) and omeprazole (20 mg gastro-resistant capsules) were obtained from Teva Ltd., Castleford, UK. Orablend^®^ was obtained from Fagron, Newcastle upon Tyne, UK. Polyfuser B, the sodium bicarbonate solution (8.4%), was purchased from Fresenius Kabi, Chesire, UK. All other reagents for HPLC analysis, including potassium phosphate dibasic (≥99.0%) and methanol CHROMASOLV^®^ (≥99.0%) were purchased from Sigma-Aldrich, Dorset, UK.

### 4.2. Pharmaceutical Analysis of Omeprazole and Amlodipine

Drug quantification was performed using reversed-phase HPLC (Agilent Technologies 1200^®^ series, Cheshire, UK). Chromatographic separation was achieved using a Spherisorb (ODS1) column (4.6 × 150 mm^2^ internal diameter with 5 µm packaging; Waters, Harrow, UK) and a SPHER5U ODS1 (1.0 × 4.6 cm) guard column (Waters, Harrow, UK) with UV detection at 238 nm for amlodipine and 302 nm for omeprazole samples, respectively. Mobile phase was a mixture of methanol and 0.05 M potassium phosphate dibasic buffer (85:15), (pH 7.4) with a run time of 8 min for omeprazole and 5 min for amlodipine. The column temperature was maintained at 20 °C and injection volume was 20 µL.

The described method for the quantification of omeprazole and amlodipine was validated [21]. Least-squares linear regression analysis and correlation analysis were performed on the triplicate calibration curves produced on each of three separate days, enabling determination of the equation of the lines and their coefficients of determination. For determination of limits of detection (LoD) and quantification (LoQ), an approach based on the standard deviation of the response and the slope of the representative calibration curve was employed.

A range of omeprazole and amlodipine standards were prepared using a solvent mixture consisting of methanol and 0.05 M potassium dibasic buffer at a ratio of 85:15 (*v/v*). The concentrations for both drugs were 20, 15, 10, 5 and 2 µg/mL. Quantification was achieved by analysis of the samples in comparison to the external standards.

### 4.3. Preparation of Omeprazole and Amlodipine Formulations

Pharmacists undertaking PhD degrees at Queen’s University Belfast and also working part-time in community practice (*n* = 20) were contacted via email in December 2011, inviting them to partake in the study. Fifteen pharmacists responded to the email, and ten were subsequently recruited. For each formulation, pharmacists were provided with a prescription for a fictional patient (Figure S1), hospital letter (Figure S2), master document describing how to formulate the products (Figure S3) and worksheet (Figure S4). All required apparatus and ingredients were provided for the pharmacists. Labels were provided for the formulations using proprietary labelling software (MPS, McLernons, Belfast, UK).

### 4.4. Drug Quantification and Stability Profile

Formulations of omeprazole and amlodipine were prepared by each pharmacist and were divided into 2 × 75 mL amber glass bottles with child resistant caps, one for storage at 4 °C and one for storage at 25 °C. At pre-determined time points, (days 0, 2, 7, 14, 21, 28 and 56), 0.05 mL samples were withdrawn, diluted in mobile phase, vortexed, filtered using a Millex-GS Filter Unit 0.22 μm (SigmaAldrich, Dorset, UK) to remove particulates and analysed by HPLC. All ten pharmacists’ formulations were assayed on day 0 to determine initial drug content and 5 of each formulation were chosen at random to assess stability over a 56-day period. The formulations were numbered and the random number function in Microsoft Excel (Microsoft Corporation, Redmond, WA, USA) was used to generate 5 numbers between 1 and 10 corresponding to the formulations chosen for the stability study.

### 4.5. Particle Size

A sample of the formulations prepared by the pharmacists was diluted in purified water (1:1000 dilution) and particle size was determined by laser diffraction using a HELOS BR laser detection sensor (Sympatec, Clausthal-Zellerfield, Germany). Five replicates of each sample were taken and measured over the range of 0.2–85 μm.

### 4.6. Antimicrobial Preservation

The efficacy of antimicrobial preservation against bacteria was assessed (days 0, 14 and 28), using a method based on the British Pharmacopeial Test (2012) for Efficacy of Antimicrobial Preservation. A series of sterile McCartney bottles containing the product to be examined were inoculated, each with a suspension of one of the test organisms to give an inoculum of 1 × 10^6^ micro-organisms per millilitre of the preparation. Products were placed in an incubator (25 °C) and protected from light. A 1 mL sample from each container was removed at zero hours and on days 14 and 28, and the number of viable microorganisms were determined by plate count. The preservative properties of the preparation were considered adequate if, in the conditions of the test, there was a fall or no increase in the number of microorganisms in the inoculated preparation after the times and at the temperatures prescribed. Acceptance criteria are defined as a 3-log reduction of bacterial load at day 14 and no increase in number of viable microorganisms compared to the previous reading at day 28.

### 4.7. Assessing Pharmacists’ Opinions

Pharmacists who participated in the study completed a self-administered questionnaire (Figure S5) regarding the preparation of the medicines. Questions addressed their experiences with extemporaneous compounding, their confidence in the product and the complexity of preparing the formulation. Space was provided for free-text responses. Responses were inputted to Microsoft Excel (Microsoft Corporation, Redmond, WA, USA) for descriptive analysis.

## 5. Conclusions

Results presented herein demonstrate that while the pharmacists who partook in the study had some or high confidence in the final extemporaneously compounded products, they reported difficulty formulating the omeprazole suspension. Analysis of drug content, stability, particle size and antimicrobial preservation highlight variation between formulations prepared by different participants. Moving forward, ways to circumvent the abovementioned difficulties are warranted. More extensive training in extemporaneous compounding could help improve the competency of pharmacists and the introduction of a single “compounding pharmacy” within different regions, where pharmacists receive continued post-graduate development and training, could be an alternative option. In addition to more extensive training, the profession would benefit from clearer guidance and continual revalidation from governing bodies, such as the PSNI, regarding the acceptable criteria for extemporaneously produced products. This should provide pharmacists with more confidence on whether their product is suitable for release to the patient. Where this is not possible, more licensed products may be required to ensure optimal pharmacological therapy, particularly for paediatric patients where the therapeutic window is narrow.

## Figures and Tables

**Table 1:** Calibration plot properties for omeprazole and amlodipine quantification method as determined by linear regression, correlation analysis and LoD/LoQ for each drug.

Drug	Slope	y-Intercept	R^2^	LoD (mcg/mL)	LoQ (mcg/mL)
**Omeprazole**	62.5	−17.28	0.998	3.47	10.50
**Amlodipine**	39.18	−8.53	0.999	0.85	2.59

**Table 2:** Day 0 drug content of the omeprazole and amlodipine formulations prepared by pharmacists.

Pharmacist ID	Omeprazole		Amlodipine	
	Concentration of Product (mg/mL)	Percentage of Theoretical Drug Content (%)	Concentration of Product (mg/mL)	Percentage of Theoretical Drug Content (%)
**1**	1.81	90.61	0.95	94.52
**2**	1.85	92.75	1.18	118.36
**3**	1.87	93.62	1.17	116.57
**4**	1.85	92.61	1.20	120.09
**5**	1.73	86.47	0.85	85.05
**6**	1.79	89.60	0.85	84.94
**7**	1.85	92.61	1.05	104.52
**8**	1.87	93.74	0.86	85.63
**9**	1.80	90.15	0.73	72.63
**10**	1.26	62.84	0.99	98.68

**Table 3:** The initial bacterial challenge in the formulations of omeprazole and amlodipine and the log reduction of bacterial load over the 28-day period.

	Mean Initial Bacterial Count		Log Reduction of Bacterial Count	
**Bacterial Species**	**Omeprazole**	**Amlodipine**	**Omeprazole**	**Amlodipine**
***Pseudomonas aeruginosa***	3.87 × 10^6^	1.88 × 10^6^	6.59	6.27
***Escherichia coli***	3.01 × 10^5^	1.88 × 10^6^	5.48	6.27
***Staphylococcus aureus***	2.37 × 10^6^	4.58 × 10^5^	6.37	5.66

**Table 4:** Statistical analysis of survey results. Wilcoxon Signed Rank Test was used to compare the medians between the two formulations. Questions on confidence were on a scale of 1–5, where 1 = no confidence to 5 = very confident. Questions on the complexity of preparing the product were on a scale of 1–5, where 1 = very easy and 5 = very difficult. IQR: interquartile range.

Statement	Median (IQR)	*p* *
Overall level of confidence extemporaneously preparing the omeprazole suspension.	3.00 (2.75–4.00)	0.16
Overall level of confidence extemporaneously preparing the amlodipine suspension.	4.00 (2.75–4.00)	
Level of complexity preparing the omeprazole suspension.	2.50 (2.00–3.25)	0.66
Level of complexity preparing the amlodipine suspension.	2.50 (2.00–3.25)	

* Wilcoxon Signed Rank Test was used to compare the medians between the two formulations. Questions on confidence were on a scale of 1–5 where 1 = no confidence to 5 = very confident. Questions on complexity of preparing the product were on a scale of 1–5 where 1 = very easy to 5 = very difficult.

## Supplementary Materials

The following are available online, Figure S1: Fictional prescription for amlodipine (A) and omeprazole (B). Figure S2: Fictional hospital letter for amlodipine (A) and omeprazole (B). Figure S3: Master document for the amlodipine (A) and omeprazole (B) suspension. Figure S4: Worksheet for amlodipine (A) and omeprazole (B). Figure S5: Survey completed by pharmacist volunteers for amlodipine (A) and omeprazole (B).
