# Supplementary material for: A Drug Content, Stability Analysis, and Qualitative Assessment of Pharmacists’ Opinions of Two Exemplar Extemporaneous Formulations
Source: Molecules. 2020 Jul 6;25(13):3078. doi: 10.3390/molecules25133078 (PMC7412138; doi:10.3390/molecules25133078)
Supplement: Supplementary file 1 [file molecules-25-03078-s001.pdf]

molecules

Supplementary Materials

A Drug Content, Stability Analysis, and Qualitative Assessment of Pharmacists' Opinions of Two Exemplar Extemporaneous Formulations

Melissa Kirkby, Kurtis Moffatt, Aoife M. Rogers, Paul J. McCague, James C. McElnay, Caoimhe Quinn, Lezley Ann McCullough, Johanne Barry and Ryan F. Donnelly *

School of Pharmacy, Medical Biology Centre, Queens University Belfast, 97 Lisburn Road, Belfast BT9 7BL, UK; m.kirkby@qub.ac.uk (M.K.); k.moffatt@qub.ac.uk (K.M.); a.rogers@qub.ac.uk (A.M.R.); p.mccague@qub.ac.uk (P.J.M.); j.mcelnay@qub.ac.uk (J.C.M.); cquinn80@qub.ac.uk (C.Q.); lmccullough01@qub.ac.uk (L.A.M.); johanne.barry@qub.ac.uk (J.B.)

* Correspondence: r.donnelly@qub.ac.uk; Tel.: +44-28-90-972-251; Fax: +44-28-90-247-794

A

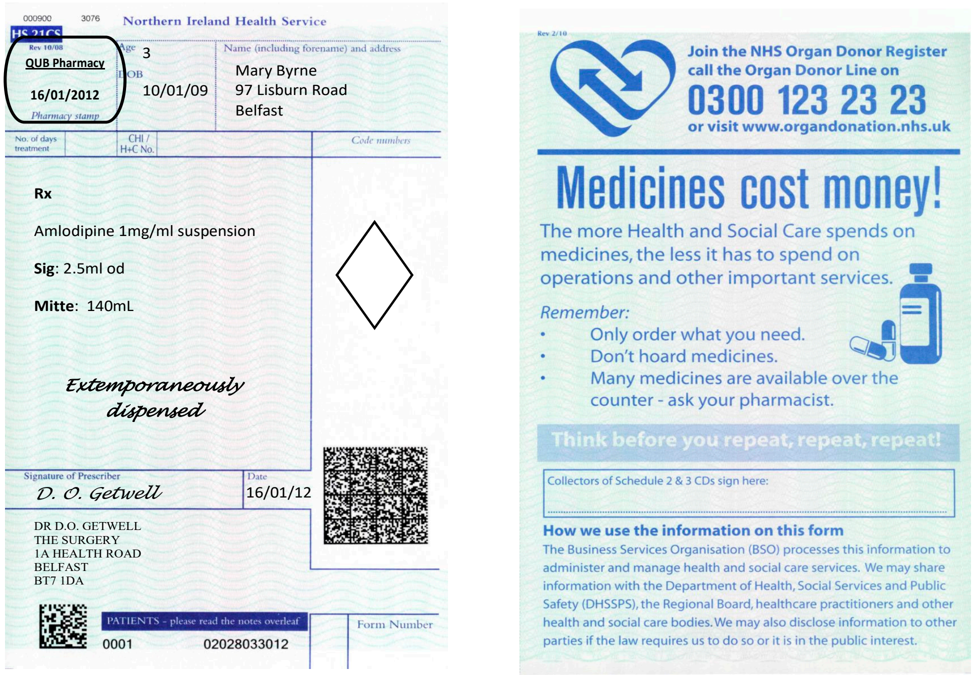

B

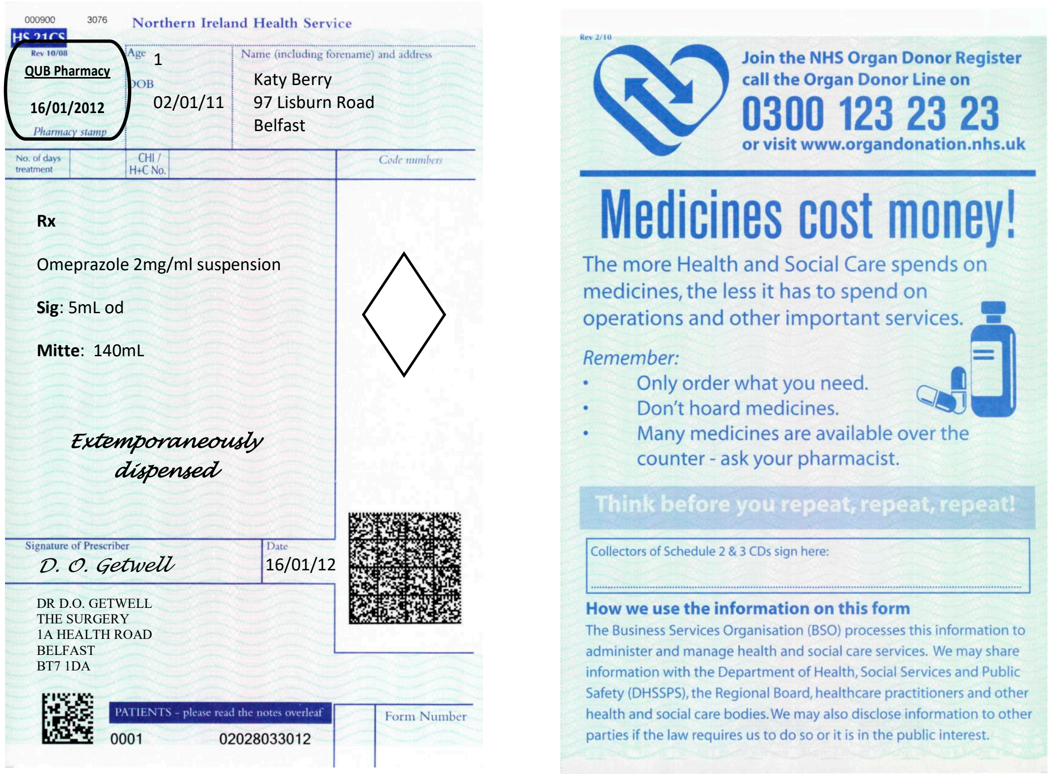

Figure S1. Fictional prescription for (**A**) amlodipine and (**B**) omeprazole.

A

The

MERNE

HOSPITALS

Merne Hospital,

16 January 2012

To: Community Pharmacist

Patient Name: Mary Byrne

Ref: *DMANAGER/FORMULAS/AML*

The above child has been discharged from our hospital. They have been commenced on amlodipine 2.5mg daily. The child is to continue treatment at home with amlodipine 1mg/ml suspension. The following is the formula used at the hospital for this suspension.

Formula:

Amlodipine 5mg tablets	x28
Water	5mL
Orablend	to 140mL

Method

1. Crush the amlodipine tablets in a mortar.
2. Add a small volume of water and make into a paste.
3. Transfer into a pre-calibrated bottle and make up to 140mL with Orablend.

Expiry: 30 days

Storage: Refrigerator

If there are any queries, please contact the Pharmacy Department, Merne Hospital.

B

The

MERNE

HOSPITALS

Merne Hospital,

16 January 2012

To: Community Pharmacist

Patient Name: Katy Berry

Ref: *DMANAGER/FORMULAS/OMEP*

The above child has been discharged from our hospital. They have been commenced on omeprazole 10mg daily via PEG tube. The child is to continue treatment at home with omeprazole 2mg/ml suspension. The following is the formula used at the hospital for this suspension.

Formula:

Omeprazole 20mg capsules	x14
Sodium bicarbonate polyfusor 8.4%	to 140mL

Method

1. Open the capsules and crush the powder in a mortar.
2. Mix with a small amount of sodium bicarbonate to form a paste.
3. Make up to volume with remaining sodium bicarbonate polyfusor.

Expiry: 4 weeks

Storage: Refrigerator

If there are any queries, please contact the Pharmacy Department, Merne Hospital.

Figure S2. Hospital letter for (**A**) amlodipine and (**B**) omeprazole.

A

Extemporaneous Dispensing Master Document: Amlodipine 1mg/ml suspension

**Name of product:**	Amlodipine 1mg/ml suspension
**Formulation:**	Amlodipine 5mg tablets x28<br>Water 5mL<br>Orablend to 140mL
**Formulation Source:**	Merne Hospital Letter; Ref: *DMANAGER/FORMULAS/AML*
**Method and Preparation:**	1. Calibrate 150ml glass amber bottle with water (to 140ml)<br>2. Crush the amlodipine tablets in a mortar.<br>3. Add a small volume of water and make into a paste, ensuring no lumps.<br>4. Transfer into the pre-calibrated bottle and make up to 140mL with Orablend.<br>5. Shake well and dispense.
**Notes/cautions:**	Store in an amber medicine bottle<br>Store in a refrigerator
**Expiry Date of finished Product:**	30 days
**Expiry Source/stability data:**	Merne Hospital – 30 days
**Master Produced by:**	Paul McCague
**Master Checked by:**	Ryan Donnelly
**Date Formula sourced/confirmed:**	06/01/2012
**Review date:**	03/02/2012

B

Extemporaneous Dispensing Master Document: Omeprazole 2mg/ml suspension

**Name of product:**	Omeprazole 2mg/ml suspension
**Formulation:**	Omeprazole 20mg capsules x14<br>Polyfusor sodium bicarbonate 8.4%<br>to 140ml
**Formulation Source:**	Merne Hospital Letter; Ref: *DMANAGER/FORMULAS/OMEP*
**Method and Preparation:**	1. Calibrate 150ml glass amber bottle with water (to 140ml).<br>2. Open the capsules and empty contents into a mortar.<br>3. Crush the contents of the capsules.<br>4. Add a small volume of sodium bicarbonate and make into a paste.<br>5. Transfer into the pre-calibrated bottle and make up to 140mL with sodium bicarbonate polyfusor.<br>6. Shake well and dispense.
**Notes/cautions:**	Store in an amber medicine bottle<br>Store in a refrigerator
**Expiry Date of finished Product:**	4 weeks
**Expiry Source/stability data:**	Merne Hospital – 4 weeks
**Master Produced by:**	Paul McCague
**Master Checked by:**	Ryan Donnelly
**Date Formula sourced/confirmed:**	06/01/2012
**Review date:**	03/02/2012

Figure S3. Master document (**A**) amlodipine and (**B**) omeprazole suspension.

A

NPA
National Pharmacy Association

Worksheet for Recording Extemporaneous Dispensing

Date: 16 January 2011

Identification no: MANAGER/FORMULAS/AML

Prescription details (item & quantity ordered):

Amlodipine 1mg/ml suspension x140mL

Formula & Calculations (including any relevant notes):

28 X 5mg = 140mg / 140ml = 1mg/ml

Source of Formula: Merne Hospital Letter; Ref: DMANAGER/FORMULAS/AML

Expiry date of finished product: 15 February 2012

Formula calculated by: Paul McCague **Formula checked by:** Ryan Donnelly

Patient name: Mary Byrne **Prescriber name:** Dr D.O. Getwell

Address: 97 Lisburn Road, Belfast **Address:** 1A Health Road, Belfast

Date of Prescription: 16/01/2012 **Date required:** 16/01/2012

INGREDIENT DETAILS

Ingredient	Manufacturer	Batch no/ Expiry Date	Quantity used	Checked by	Pharmacist signature
Amlodipine 5mg tablets	Teva	1H42UK / 07/2016	28		
Ora-Blend SF	Paddock Lab.	1145251 / 04/2013	to 140ml		

Final check Signature: **Copy of label:**

B

National Pharmacy Association

Worksheet for Recording Extemporaneous Dispensing

Date: 16 January 2011

Identification no: MANAGER/FORMULAS/OMEP

Prescription details (item & quantity ordered):

Omeprazole 2mg/ml suspension x140mL

Formula & Calculations (including any relevant notes):

14 X 20mg = 280mg in 140mL
=2mg/ml

Omeprazole 20mg capsules x14

Polyfusor sodium bicarbonate 8.4% to 140ml

Source of Formula: Merne Hospital Letter; Ref: DMANAGER/FORMULAS/OMEP

Expiry date of finished product: 13 February 2012

Formula calculated by: Paul McCague **Formula checked by:** Ryan Donnelly

Patient name: Katy Berry **Prescriber name:** Dr D.O. Getwell

Address: 97 Lisburn Road, Belfast **Address:** 1A Health Road, Belfast

Date of Prescription: 16/01/2012 **Date required:** 16/01/2012

INGREDIENT DETAILS

Ingredient	Manufacturer	Batch no/ Expiry Date	Quantity used	Checked by	Pharmacist signature
Omeprazole 20mg capsules	Teva	LC08069 / 05/2014	14		
Polyfusor B Sodium Bicarbonate 8.4%	Fresenius Kabi	11B212 / 09/2012	to 140ml		

Final check Signature: **Copy of label:**

Figure S4. Worksheet for (**A**) amlodipine and (**B**) omeprazole.

A

Extemporaneous Preparation of Medicines &

Manipulation of Solid Dosage Forms

Question 1*: *Omeprazole Suspension in Polyfusor Sodium Bicarbonate Solution 8.4% from the solid dosage form (capsule).

(a) Had you ever prepared this omeprazole formulation in the past?

Yes	No
☐	☐

(b) What was your overall level of confidence in extemporaneously preparing the omeprazole suspension?

No confidence	Low Confidence	Some confidence	High confidence	Very confident
☐	☐	☐	☐	☐

(c) How would you rate the level of complexity making this suspension:

Very Easy	Easy	Neutral	Difficult	Very difficult
☐	☐	☐	☐	☐

Please make any comments you have on the preparation of omeprazole suspension.

____

____

____

____

B

***Question 2*: Amlodipine Suspension in Orablend® from the solid dosage form (tablet).**

(a) Had you ever prepared this amlodipine formulation in the past?

Yes	No
☐	☐

(b) What was your overall level of confidence in extemporaneously preparing the amlodipine suspension?

No confidence	Low Confidence	Some confidence	High confidence	Very confident
☐	☐	☐	☐	☐

(c) How would you rate the level of complexity making this suspension:

Very Easy	Easy	Neutral	Difficult	Very difficult
☐	☐	☐	☐	☐

Please make any comments you have on the preparation of amlodipine suspension.

____

____

____

____

Figure S5. Survey completed by pharmacist volunteers for (**A**) omeprazole and (**B**) amlodipine.
